# Relationship between the inner setting of CFIR and the delivery of the Healthy School Recognized Campus initiative: a mixed-methods analysis

**DOI:** 10.1186/s43058-024-00627-3

**Published:** 2024-09-04

**Authors:** Allyson Schaefers, Lucy Xin, Paula Butler, Julie Gardner, Alexandra L. MacMillan Uribe, Chad D. Rethorst, Laura Rolke, Rebecca A. Seguin-Fowler, Jacob Szeszulski

**Affiliations:** 1 Institute for Advancing Health Through Agriculture (IHA), Texas A&M University, 17360 Coit Rd, Dallas, TX 75252 USA; 2 Texas A&M AgriLife Extension, 600 John Kimbrough Boulevard, College Station, TX 77843 USA; 3 Texas 4-H Youth Development, 1470 William D Fitch Parkway, College Station, TX 77845 USA; 4 Department of Population and Health Sciences, Duke University School of Medicine, 215 Morris Street, Durham, NC 27701 USA; 5 Institute for Advancing Health Through Agriculture (IHA), Texas A&M University, 1500 Research Parkway, Centeq Building B, College Station, TX 77845 USA

**Keywords:** Physical activity, Exercise, Eating behaviors, Implementation, Dissemination, Social environment

## Abstract

**Introduction:**

Healthy School Recognized Campus (HSRC) is a Texas A&M AgriLife Extension initiative that promotes the delivery of multiple evidence-based physical activity and nutrition programs in schools. Simultaneous delivery of programs as part of HSRC can result in critical implementation challenges. The study examines how the inner setting constructs from the Consolidated Framework for Implementation Research (CFIR) impact HSRC program delivery.

**Methods:**

We surveyed (*n* = 26) and interviewed (*n* = 20) HSRC implementers (*n* = 28) to identify CFIR inner setting constructs related to program acceptability, appropriateness, and feasibility. Using a concurrent mixed-methods design, we coded interviews using the CFIR codebook, administered an inner setting survey, tested for relationships between constructs and implementation outcomes via chi-square tests, and compared quantitative and qualitative results.

**Results:**

Stakeholders at schools that implemented one program vs. more than one program reported no differences in acceptability, appropriateness, or feasibility outcomes (p > .05); however, there was a substantial difference in reported program minutes (1118.4 ± 951.5 vs. 2674.5 ± 1940.8; p = .036). Available resources and leadership engagement were related to HSRC acceptability (r = .41; p = .038 and r = .48; p = .012, respectively) and appropriateness (r = .39; p = .046 and r = 0.63; p = .001, respectively). Qualitative analyses revealed that tangible resources (e.g., curriculum, a garden) enabled implementation, whereas intangible resources (e.g., lack of time) hindered implementation. Participants also stressed the value of buy-in from many different stakeholders. Quantitative results revealed that implementation climate was related to HSRC acceptability (r = .46; p = .018), appropriateness (r = .50; p = .009), and feasibility (r = .55; p = .004). Learning climate was related to HSRC appropriateness (r = .50; p = .009). However, qualitative assessment of implementation climate subconstructs showed mixed perspectives about their relationship with implementation, possibly due to differences in the compatibility/priority of different programs following COVID-19. Networks/communication analysis showed that schools have inner and outer circles of communication that can either benefit or hinder implementation.

**Conclusion:**

Few differences were found by the number of programs delivered. Implementation climate (i.e., compatibility, priority) and readiness for implementation (i.e., resources and leadership engagement) were important to HSRC implementation. Strategies that focus on reducing time-related burdens and engaging stakeholders may support HSRC’s delivery. Other constructs (e.g., communication, access to knowledge) may be important to the implementation of HSRC but need further exploration.

**Supplementary Information:**

The online version contains supplementary material available at 10.1186/s43058-024-00627-3.

Contributions to the literature

- The simultaneous delivery of multiple evidence-based programs can result in critical implementation challenges.
- Mixed methods approaches can quantify the magnitude of association between implementation barriers and outcomes while also providing a nuanced understanding of how implementation strategies can address those barriers.
- Readiness for implementation (i.e., resources and leadership engagement) is important to the delivery of multiple programs as part of the Healthy School Recognized Campus initiative.
- Implementation strategies that focus on reducing time-related burdens and engaging stakeholders may be beneficial for supporting the Healthy School Recognized Campus initiative.

## Introduction

Reviews of school- and evidence-based programs for improving physical activity and nutrition show reductions in obesity prevalence by up to 8% [1–9]. Many models and frameworks (e.g., Whole School, Whole Community, Whole Child) emphasize the delivery of multiple programs [10–12], which can result in critical implementation challenges (e.g., limited resources and communication barriers) [13–15]. Despite challenges, most schools deliver numerous programs within the same school day or year. Thus, it is important to understand how to support simultaneous implementation of programs.

The Consolidated Framework for Implementation Research (CFIR) examines five domains that affect implementation: intervention characteristics, outer setting, inner setting, characteristics of individuals, and implementation processes [16–19]. Although all CFIR constructs affect implementation, within schools, the inner setting has a major impact on program delivery and may be critically important when assessing the simultaneous delivery of multiple evidence-based programs [20]. Furthermore, implementation strategies that target the inner setting have the potential to improve delivery of multiple programs [21, 22].

CFIR is commonly used to assess barriers/facilitators related to the implementation of school-based programs [20, 23–25]. One systematic review identified several inner setting factors – administrative support, staff engagement, and access to resources – as central to supporting implementation [20]. Qualitative findings highlighted important lessons including: (1) conduct a readiness assessment, (2) identify wellness champions, (3) build on existing curricula, and (4) conduct ongoing training [20]. However, this study broadly focused on all CFIR constructs. Thus, limited inner setting information was provided.

Qualitative methods provide context, whereas quantitative methods describe the magnitude of a relationship between program barriers/facilitators and implementation outcomes [26–29]. Mixed methods approaches that bridge quantitative and qualitative methods can generate new insights [28]. For example, one mixed methods study quantified inner setting barriers to program implementation – lack of time/resources, staff buy-in, administrator support – but also found that outer setting educators could be utilized to overcome those barriers [30]. This type of nuanced understanding of implementation processes is especially important with increased complexity, such as when programs are concurrently delivered.

To elucidate inner setting factors related to concurrently implementing school-based physical activity and nutrition programs, we conducted a mixed methods study that examined the implementation of a Texas A&M AgriLife Extension initiative, Healthy School Recognized Campus (HSRC).

## Methods

### Study design and participants

HSRC is a program that rewards schools for delivering health programs, including an evidence-based school-wide walking program, one additional adult program, and one additional youth program. Outside of the evidence-based walking program, schools fill out an application at the beginning of the school year to choose between evidence-based programs and ones easier to implement (full list of programs and their evidence base can be found at https://texas4h-hsrc.com/). Programs are delivered by Extension agents (i.e., agents) with the support of school staff. School staff often only implement one program, while agents implement two or more at each school. Schools that complete programs receive a banner and a proclamation at a local school board meeting. Not all schools that enroll in HSRC become recognized, only those that complete selected programs by a specific deadline (i.e., May 1).

We utilized a cross-sectional, concurrent mixed methods design consisting of surveys and/or interviews grounded in CFIR (spring of 2022). Integration of methods occurred during the design (i.e., aligning qualitative and quantitative constructs) and data analysis (i.e., comparing and contrasting results) phases and are reported following both guidelines (Supplemental Table 1). Texas A&M’s Institutional Review Board approved this study (IRB2022-0390 M).

We recruited a convenience sample of HSRC program implementers (e.g., teachers, agents) from eight elementary schools in rural East Texas. Participants completed a survey, interview, or both based on their interest. Inclusion criteria were being at least 18 years old and speaking English.

### Surveys and interviews

When designing the survey and interview guide, we aligned CFIR constructs by selecting and using existing instruments that contained as many inner setting constructs as possible. We assessed inner setting constructs (Definitions—Table 1) and implementation outcomes (i.e., acceptability, appropriateness, feasibility) via validated survey measures that used Likert scales (strongly disagree to strongly agree) [31, 32]. Program minutes were assessed via three questions that asked, “How many [weeks, days/week, and minutes/day] did students participate in [program]?” Surveys (*n* = 26), collected using REDCap, lasted about 20–30 min. Participants received a $20 gift card.

**Table 1:** CFIR Inner Setting Construct Definitions

A. Structural Characteristics		The social architecture, age, maturity, and size of an organization
B. Networks and Communications		The nature and quality of webs of social networks and the nature and quality of formal and informal communications within an organization
C. Culture^a^		Norms, values, and basic assumptions of a given organization
D. Implementation Climate^a^		The absorptive capacity for change, shared receptivity of involved individuals to an intervention, and the extent to which use of that intervention will be rewarded, supported, and expected within their organization
	D1. Tension for Change	The degree to which stakeholders perceive the current situation as intolerable or needing change
	D2. Compatibility	The degree of tangible fit between meaning and values attached to the intervention by involved individuals, how those align with individuals’ own norms, values, and perceived risks and needs, and how the intervention fits with existing workflows and systems
	D3. Relative Priority	Individuals’ shared perception of the importance of the implementation within the organization
	D4. Organizational Incentives and Rewards	Extrinsic incentives such as goal-sharing awards, performance reviews, promotions, and raises in salary, and less tangible incentives such as increased stature or respect
	D5. Goals and Feedback	The degree to which goals are clearly communicated, acted upon, and fed back to staff, and alignment of that feedback with goals
	D6. Learning Climate^a^	A climate in which: a) leaders express their own fallibility and need for team members’ assistance and input; b) team members feel that they are essential, valued, and knowledgeable partners in the change process; c) individuals feel psychologically safe to try new methods; and d) there is sufficient time and space for reflective thinking and evaluation
E. Readiness for Implementation		Tangible and immediate indicators of organizational commitment to its decision to implement an intervention
	E1. Leadership Engagement^a^	Commitment, involvement, and accountability of leaders and managers with the implementation
	E2. Available Resources^a^	The level of resources dedicated for implementation and on-going operations, including money, training, education, physical space, and time
	E3. Access to Knowledge and Information	Ease of access to digestible information and knowledge about the intervention and how to incorporate it into work tasks

^a^ Included in the Fernadez et al. 2018 inner setting survey measure. Culture effort and culture stress were also included

Utilizing the CFIR website, two researchers (JS, AS) developed an interview guide that asked questions about how inner setting constructs affected program delivery: (a) school leaders, staff, and students’ perceptions; “What do you think are leaderships’ impressions of the HSRC program?” (b) schools’ characteristics (e.g., organizational structure, space); “How do you think the physical design of the school – playgrounds, gyms – affected the implementation?”(c) culture (i.e., shared beliefs and values); “How do you think the culture of your school affected the implementation of HSRC?”(d) resources; “What resources are available at your school to implement HSRC?” Interviews were conducted at elementary schools (*n* = 13) or online (*n* = 7), audio-recorded, and lasted 30–60 min. Participants received a $50 gift card.

We used NVivo to transcribe and review audio files. Applying a directed content analysis and iterative categorization approach [33, 34], we used a priori codebook, based on CFIR inner setting constructs, to deductively code transcripts. Two researchers (AS, LR) independently coded four transcripts and discussed line-by-line discrepancies to consensus. One researcher (AS) coded the remaining 16 transcripts. Two researchers (AS, LX) independently read code queries noting important findings, summarizing constructs, and highlighting quotes. Three researchers (AS, LX, JS) constructed themes synthesizing the results. We compared themes between stakeholders that implemented one program vs. more than one program.

### Statistical methods

We scored CFIR inner setting constructs and implementation outcomes using established protocols [31, 32]. For constructs missing less than 75% of the data (seven total responses), we imputed missing values as the average of all other responses for that construct. We calculated program minutes as the days/week, times total weeks, times average session length, summed across all programs. We conducted descriptive statistics and chi-square tests, in SPSS 27, to assess relationships between CFIR constructs and implementation outcomes. We also compared the direction of quantitative analysis (i.e., positive or negative association) with findings from the qualitative analysis, when applicable.

## Results

Five of the eight schools completed 2 evidence-based programs, but all schools completed at least one (Table 3). Most participants (*n* = 28; *n* = 2 interview only, *n* = 8 survey only, *n* = 18 both) were female and classroom teachers (Table 2). Schools (*n* = 8) had on average 327.1 ± 158.3 students (14.9% Black/African American; 30.1% Hispanic; and 50.0% White), included 75.4% economically disadvantaged students, were Title I (100%), and on average implemented 2.1 ± 1.2 programs (Table 3). When comparing stakeholders at schools that implemented one program vs. more than one program, there were no differences in qualitative themes, acceptability, appropriateness, or feasibility outcomes (p > 0.05); however, there was a substantial difference in reported program minutes (1,118.4 ± 951.5 vs. 2,674.5 ± 1,940.8; p = 0.036).

**Table 2:** Descriptive characteristics of participants

	Survey Only *n* =8	Both *n* = 18
Age, years (*M* ± *SD*)	44.6 ± 6.2	44.1 ± 11.2
Female (%)	87.5	94.1
Role (%)		
Cooperative Extension	12.5	22.2
Classroom/Physical Education Teacher	62.5	44.4
School Administrator	12.5	11.1
Other	12.5	22.2
Years Worked at School (*M* ± *SD*)	7.0 ± 7.8	7.8 ± 7.4
Years Worked in Education (*M* ± *SD*)	17.0 ± 5.6	14.9 ± 10.6

**Table 3:** School-level participation in healthy school recognized campus programs and implementation outcomes

School Number	Walk Across Texas^a^ [35, 36]	Health, Food, Fun & Fitness^a^ [37, 38]	Learn, Grow, Eat, and Go^a^ [36, 39]	Path to the Plate	4-H Food and Nutrition Opportunities	Total Programs	Total Evidence-based Programs	Acceptability	Appropriateness	Feasibility	Program Minutes
1			X			1	1	4.7	4.7	4.7	968.8
2			X			1	1	4.1	4.0	4.4	870.9
3			X			1	1	4.6	4.5	4.5	1,308.8
4	X		X			2	2	4.3	4.0	4.2	2,104.7
5	X	X				2	2	5.0	5.0	5.0	1,470.0
6	X		X	X	X	4	2	5.0	4.1	3.1	3,555.0
7	X		X	X	X	4	2	4.4	4.5	4.4	1,315.5
8	X		X			2	2	4.1	4.1	4.0	2,891.3

Note—^a^Evidence-based program. Program descriptions available at https://texas4h-hsrc.com/

### Implementation climate

Quantitatively, implementation climate was associated with the acceptability, appropriateness, and feasibility of HSRC (Table 4). The only implementation climate subconstruct measured was *learning climate*, which was associated with appropriateness, but not discussed in interviews.

**Table 4:** Association between inner setting constructs and implementation outcomes

CFIR Constructs	Acceptability		Appropriateness		Feasibility		Total program minutes	
	*r*	*p-value*	*r*	*p-value*	*r*	*p-value*	*r*	*p-value*
Culture^a^	0.199	0.329	0.260	0.200	0.083	0.687	0.094	0.642
Culture Stress^a^	-0.359	0.072	-0.366	0.066	-0.224	0.272	-0.114	0.573
Culture Effort^a^	0.333	0.096	0.360	0.071	-0.031	0.881	0.170	0.395
Implementation Climate^b^	**0.462**	**0.018**	**0.501**	**0.009**	**0.545**	**0.004**	0.162	0.418
Learning Climate^b^	0.253	0.213	**0.503**	**0.009**	0.298	0.140	-0.038	0.850
Readiness for Implementation								
Leadership Engagement^c^	**0.408**	**0.038**	**0.394**	**0.046**	0.007	0.973	0.540	0.789
Available Resources^c^	**0.483**	**0.012**	**0.633**	**0.001**	0.213	0.297	0.131	0.516

^*^Bolded values are statistically significant (*p-*value < 0.05); a – summarized as culture in qualitative findings; b – summarized as climate in qualitative findings; c – summarized as readiness for implementation in qualitative findings

Qualitatively, within implementation climate, interviewees shared different perspectives on schools’ *tension/need for change* (i.e., adoption of HSRC) based on the community’s and students’ needs (Table 5—Quote 1). An Extension agent (i.e., agent) also noted that some teachers would become interested in programs after seeing the positive effects, such as students keeping each other and teachers accountable for health behaviors.

**Table 5:** Quotes for CFIR Construct: Implementation Climate

Quote 1	“…a lot of them [students] are going home having to prepare meals for their younger siblings and different things like that… Our staff show up and do what they need to do so that students can learn, you know, and be able to take it back home.”
	**-#12 Teacher (2 programs)**
Quote 2	“This state just changed the TEKs for physical education…to have health included. So, I just kind of push it to them that way like this would help put health into your curriculum and PE for these elementary students. And here's the TEKs for the programs that I’m providing. And would you be interested in scheduling me to come?”
	**-#16 Extension agent (3 programs)**
Quote 3	“The biggest barrier? Just…timing of it and getting it started and seeing how it can fit into, you know, what I'm doing currently in the classroom.”
	**-#10 Teacher (2 programs)**
Quote 4	“…in the spring sometimes it's harder to get in because [the schools are] really like crunch time with the STAAR tests. So, I was kind of working around that and the STAAR test schedule, that's probably more challenging.”
	**-#19 Extension agent (3 programs)**
Quote 5	“pre-K is just much more flexible, I think. So, they don't have all these requirements that they have to meet.”
	**-#17 Extension agent (2 programs)**
Quote 6	“I do a lot there [school] and have a lot of support from the SHAC and through other people, but whenever I presented this at the SHAC meeting they’re all like, “Oh yeah this all sounds great, like to do one day” or they kind of just say like, “Oh, we like these pieces of it.” And but they didn't really get the whole, “Let’s do this in a year or so, we get this recognition.” It just seemed like too much for them.”
	**-#14 Extension agent (1 program)**
Quote 7	“And it's very hard with fifth graders because we can't hold athletics over their head… But my kindergarten through third grade PE teacher, she's like, “They do the president fitness thing…” When they start with the high school coaches, it's a little different… They learn about all the little sports and kind of preparing them to get ready.”
	**-#9 Principal (2 programs)**
Quote 8	“…the cafeteria staff prepare them and include them with their lunch or whatever. And that just was not it, it just sounded like work to them.”
	**-#14 Extension agent (1 program)**

Most interviewees valued the *compatibility* of HSRC programs with the schools’ current curriculums, as they were aligned with the state educational standards (Texas Essential Knowledge & Skills [TEKS]). One agent used the fact that HSRC programs were TEKS-aligned to promote adoption (Table 5—Quote 2). Although programs aligned with state learning requirements, the programs’ timelines did not necessarily match teachers’ scheduled lesson plans or state testing schedules (Table 5 – Quotes 3 & 4). However, an agent noted that not all grade levels participate in state tests (Table 5 – Quote 5).

Not all school stakeholders viewed HSRC as a *relative priority*. When HSRC was presented to the School Health Advisory Council (SHAC), they only expressed interest in certain aspects of the initiative and were apprehensive about implementing the full program (Table 5 – Quote 6). Due to the timing of these interviews, COVID-19 restrictions still limited most in-person activities, resulting in HSRC being ranked as a lower priority. Many interviewees also stated that competing priorities (e.g., staff responsibilities, school sports) took precedence (Table 5—Quote 7 & 8). *Organizational incentives and rewards* and *goals and incentives* were not often discussed.

### Readiness for implementation

Quantitatively, leadership engagement and available resources were associated with acceptability and appropriateness (Table 4). Access to knowledge and information was not measured.

Qualitatively, schools were generally optimistic and demonstrated indicators of their readiness for implementing HSRC. Related to *leadership engagement*, school leadership supported schools’ participation in HSRC, however, engagement varied. Sometimes, principals knew that staff implemented HSRC, but they did not involve themselves or push for more school-wide programming (Table 6—Quote 1). Some principals’ involvement stopped short ( after providing approval, whereas some principals participated in the programs themselves (e.g., team captain for the walking challenge) or sought out new health-related opportunities (Table 6—Quote 2). Interviewees expressed that endorsement from leadership was imperative to launch HSRC, and leadership approval usually guaranteed implementation (Table 6 – Quote 3).

**Table 6:** Quotes for CFIR Construct: Readiness for Implementation

Quote 1	“The principal is not really involved with [the program]. She never has been too interested, but the counselor, she's always on board and willing to help.”
	**-#14 Extension agent (1 program)**
Quote 2	“The principal at [one school], is very open to trying new programs and doing anything about bringing in outside ideas and programs and working with different organizations”
	**-#17 Extension agent (2 programs)**
Quote 3	“Once principals got behind [the HSRC initiative] and said, ‘I want this to happen.’ Then it’s going to happen.”
	**-#17 Extension agent (2 programs)**
Quote 4	“I mean, our school is great about resources. If I need it, they get it. And if I don't know how to get it or have a question about it, the Extension office gets it for us.”
	**-#13 Teacher (1 program)**
Quote 5	“I don't have volunteers…I could do so much more if I had the time and the resources and the volunteers available to do additional programming…I really just struggle with having the time to find volunteers and train volunteers. We don't have a lot of organizations like that, that would commit to a 10-week program.”
	**-#16 Extension agent (3 programs)**
Quote 6	“The biggest barrier, I think, would just be time. Teachers just are already so overwhelmed with everything, with the mandates from the state.”
	**-#7 Principal (data not available)**
Quote 7	“[The Extension agents] are fantastic and I love them. There's never been a time that I've asked for something that they could not help me with, whether that was the dreaded technology. [The Extension agent] was on the phone with me the whole time working through that. I just cannot tell you how supportive they have been…Anything I've needed…whether it's on a weekend for a competition or anything.”
	**-#13 Teacher (1 program)**
Quote 8	“Since [the schools] were already on board, I pretty much take them everything. They don't have to go online to access any of those resources. I pretty much try to make it as easy and simple as possible. So they don't have to do that.”
	**-#15 Extension agent (data not available)**
Quote 9	“…it was overwhelming at first, I guess, when [the Extension agent] sent me the email, but then once she explained it all, everything was pretty self-explanatory and everybody figured it out pretty quick.”
	**-#8 Nurse (1 program)**

For *available resources*, there appeared to be two categories – tangible and intangible. Tangible resources were readily available for program implementation, either through the school, the agent, or community donations (Table 6—Quote 4). From the school’s perspective, the agent provided most, if not all, of the materials needed for HSRC (e.g., marketing materials, curricula). From the agent’s perspective, schools already had most of the items that they needed (e.g., pencils and printing capabilities). For items that needed to be purchased, agents sought out small grants, Extension funding, or local community donations.

Intangible resources included volunteer support and time, but it was a lack of these resources that seemed to negatively affect program implementation. Many interviewees recognized the positive impact volunteers had on implementation. Volunteers helped repair the facilities (e.g., gardens), taught program lessons, and even provided funding. However, agents and school staff both mentioned the need for more volunteers (Table 6—Quote 5). Interviewees also discussed how the time needed for orienting schools, lesson planning, and teaching program lessons served as barriers to implementation. Principals and agents commented on time as a barrier more than school staff (Table 6—Quote 6).

Regarding *access to knowledge*, most school staff stated that their agent provided them with the information needed for program implementation. Many highlighted the helpfulness and accessibility of their agent (Table 6—Quote 7), which agents also described as one of their own goals (Table 6—Quote 8). For this reason, programs that initially felt overwhelming became easier to implement (Table 6—Quote 9). For agents, access to knowledge was less readily available. One agent discussed how new agents faced challenges in implementing HSRC due to inaccessible program information scattered across platforms, inefficient communication with schools, and uncertainties about how to implement HSRC. From the agents’ perspective, Extension leadership served as the main source of knowledge for HSRC. One agent discussed connecting with other agents about questions, but they also recommended setting up more frequent meetings. Similarly, a couple of school staff suggested having meetings or a discussion board with implementers from different schools to share experiences and ideas.

### Culture, structural characteristics and networks & communications

Quantitatively, culture effort, but not culture or culture stress, was marginally related to HSRC’s acceptability and appropriateness (p < 0.10; Table 4). Structural characteristics and networks and communications were not included in the survey.

Qualitatively for *culture*, many implementers highlighted how living in a small community fostered an emphasis on agricultural programming (Table 7—Quote 1). Many also commented on the positive culture of support that they saw for HSRC (Table 7—Quote 2). Principals highlighted the positivity and willingness of teachers to implement HSRC (Table 7 – Quote 3), compared to teachers who identified the principal as the driving force (Table 7 – Quote 4).

**Table 7:** Quotes for CFIR Constructs: Culture, Structural Characteristics and Networks & Communications

Quote 1	“We are a very rural community, and the mindset is already there for pro gardening, pro [agriculture], pro, you know, working outside. So, like I said, the mindset is already there.”
	**-#10 Teacher (2 programs)**
Quote 2	“[The team is] really open to new ideas that are going to support the needs of the students.”
	**-#12 Teacher (2 programs)**
Quote 3	“I think as long as you have a positive culture and a culture of lifelong learners and constantly growing…implementing something like [these programs] will get done.”
	**-#7 Principal (data not available)**
Quote 4	“… our principal’s willingness to jump in and let us do these things that I’ve shown interest in was just phenomenal.”
	**-#5 Teacher (2 programs)**
Quote 5	“I feel like it's small enough of a community that I could reach out to [get] help from anyone… just everyone's close-knit and knows one another.”
	**-#5 Teacher (2 programs)**
Quote 6	“The size of the school is important because I don't have any volunteers…each class in [the school] has an average of 100 students per class. And for me to go in a forty-five minute lunch period where I might have…back to back classes of 50… I would spend an entire day and then the financial—it would cost a lot more.”
	**-#16 Extension agent (3 programs)**
Quote 7	“… you don’t see people walking blocks. I mean, they drive…everywhere…there’s not really a place that you would walk a lot in the town.”
	**-#20 Nurse (2 programs)**
Quote 8	“I'm finding somebody… I have so many hats and there are some things, if I can get somebody else to do it, it would be great. Some things I just make the decision. ‘Oh 5th grade is going to do this.’”
	**-#9 Principal (2 programs)**
Quote 9	“I have my contact at [one school]. She is so personable and just like, she’s got kids in the school and can talk to them…and she did this phenomenal job getting those moms that would drop their kids off from school.”
	**-#19 Extension agent (3 programs)**
Quote 10	“I know [the students] are going home and talking about [the program] because the parents are asking, ‘What is this that they’re talking about?’”
	**-#7 Principal (data not available)**

For *structural characteristics*, most interviewees stated that being in a smaller school, compared to a larger school, was beneficial for implementation (Table 7—Quote 5). Agents also reported that implementing the program in larger schools was more difficult due to their own time constraints, financial barriers, and lack of volunteers (Table 7—Quote 6). However, being in a rural setting negatively impacted schools’ ability to implement some programs, such as the walking challenge (Table 7—Quote 7).

For *communication*, there seemed to be both an inner and outer circle. The inner circle, involved with HSRC implementation, was typically made up of a school administrator, agent, and a few staff. The agent was generally the HSRC expert, and they would share program information. In some instances, the principal decided on which staff would facilitate communication with the agent (Table 7—Quote 8). In other cases, the agent worked with an existing contact or friend (Table 7—Quote 9). Most interviewees stated that only school staff involved in HSRC – the inner circle – knew enough about the programs to talk about them. The outer circle – school staff who did not implement HSRC and parents – might have noticed the HSRC activities but were unaware of the larger initiative. Parents’ main source of HSRC information came from their students (Table 7—Quote 10). Agents expressed feeling like they were also part of the outer circle because they were unaware of everything happening at the school (e.g., school culture, competing priorities).

## Discussion

Three schools reported implementing a single HSRC program, whereas five schools reported implementing multiple HSRC programs. However, few differences were found in our results by the number of programs delivered. Furthermore, many teachers did not know about other programs being delivered simultaneously, and as a result interviewees did not discuss related challenges. Thus, many of our identified implementation barriers match the previous literature on barriers to the implementation of single program [9, 10, 16], although our qualitative findings provide additional context.

Several inner setting constructs, including resources, leadership, and communication seemed to be more important than other constructs, such as culture. Although implementation climate appeared important in the quantitative analysis, in the qualitative analysis, subconstructs from implementation climate (e.g., compatibility, priority) presented conflicting findings that may have been related to implementation right after COVID-19. In general, qualitative analyses added depth that helped us understand better how inner setting constructs function as a part of the multicomponent HSRC initiative.

Within readiness for implementation, both leadership engagement and available resources were important for delivery; however, interviews provided deeper insight. Resources for HSRC fell into two categories – tangible and intangible. Tangible resources were readily available, whereas time, an intangible resource, was a barrier to HSRC delivery. Our study adds to the current literature [9, 10, 16], as it demonstrates that time affects the acceptability and appropriateness of delivering multiple school-based physical activity and nutrition programs but may not relate to program minutes. Implementation strategies that reduce time-related burdens and improve time management strategies may support future implementation efforts.

Schools involved many stakeholders in HSRC delivery, and each stakeholder had different roles/responsibilities, which hindered agents from getting HSRC adopted. Agents described needing to customize their approach for each school, including working through existing connections (e.g., friends) to get buy-in. Previous research has emphasized the role of leadership as gatekeepers and the need for their engagement and support for program adoption [20, 24, 40]. We found that principals also choose which teachers implement programs, these teachers were not always aware of all the programs being delivered at their schools. Furthermore, some principals stayed involved with the programs once adopted, whereas other principals passed those responsibilities to school staff. More research is needed to understand the most effective role of school leadership: a one-time authority figure or an ongoing facilitator.

Finally, we found that schools had an inner and outer circle of communication, and agents felt that they belonged to both circles, which made it difficult to bridge gaps between all stakeholders involved with program delivery. Previous research found that strong communication between leadership and other program stakeholders leads to successful implementation, whereas ineffective communication inhibits implementation [24]. Additional research is needed to better understand the purpose of having an inner and outer communication circle and to determine if there is a need to bridge the gap between the two groups, potentially via more centralized communication systems.

### Limitations

Not all programs available as part of HSRC are evidence-based. However, easier to implement programs are included as a way for schools to work up to more complex (e.g., longer) evidence-based interventions. As few validated instruments to measure CFIR’s inner setting exist, all constructs measured in the qualitative analysis could not be measured through the survey. Some sections of interviews were coded as multiple constructs, but we reached a consensus on where best to discuss them within this paper. A consensus was also developed during the coding process instead of calculating inter-rater reliability. The CFIR team has recently added new constructs to the inner setting, which do not have validated measures (e.g., mission alignment). These constructs may also be important to consider and should be tested in future studies in relationship to additional implementation outcomes (e.g. adherence/fidelity). The small sample size and inclusion of only rural schools may limit this study’s generalizability to larger and more urban schools. Not everyone completed both the interview and survey, and as a result, qualitative and quantitative perspectives may not be perfectly aligned. However, a majority did complete both activities (64%).

## Conclusions

This study utilized the CFIR framework in conjunction with a mixed methods approach to evaluate barriers and facilitators for the simultaneous implementation of multiple school-based physical activity and nutrition programs as part of HSRC. Few differences were found by the number of programs delivered or in comparison to previous studies evaluating the implementation of a single program. However, our analyses found that readiness for implementation (i.e., resources and leadership engagement) was vital to successful program implementation. Other constructs may need more research. Future research can use these findings to begin to develop implementation strategies that support the successful implementation of HSRC or other initiatives that aim to implement multiple concurrent physical activity and nutrition programs.

## Supplementary Information

Supplementary Material 1

## Data Availability

The datasets used and/or analyzed during the current study are available from the corresponding author on reasonable request.

## Abbreviations

CFIR: Consolidated Framework for Implementation Research
CPSTF: Community Preventive Services Task Force
HSRC: Healthy School Recognized Campus
REDCap: Research Electronic Data Capture
SHAC: School Health Advisory Council
TEKS: Texas Essential Knowledge & Skills
